# Cardiac tissue engineering: a reflection after a decade of hurry

**DOI:** 10.3389/fphys.2014.00365

**Published:** 2014-09-23

**Authors:** Valentina Di Felice, Rosario Barone, Giorgia Nardone, Giancarlo Forte

**Affiliations:** ^1^Department of Experimental Biomedicine and Clinical Neurosciences, University of PalermoPalermo, Italy; ^2^Department of Stress Biology, Epigenetics and Biomarkers, Euro-Mediterranean Institute of Science and Technology (IEMEST)Palermo, Italy; ^3^Integrated Center for Cell Therapy and Regenerative Medicine (ICCT), International Clinical Research Center, St. Anne's University HospitalBrno, Czech Republic

**Keywords:** cardiac progenitor cells, proto-tissues, heart regeneration, tissue engineering, scaffolds, biomaterials

The heart is a perfect machine whose mass is mainly composed of cardiomyocytes, but also fibroblasts, endothelial, smooth muscle, nervous, and immune cells are represented. One thousand million cardiomyocytes are estimated to be lost after myocardial infarction, their loss being responsible for the impairment in heart contractile function (Laflamme and Murry, 2005).

The potential success of cardiac cell therapy relies almost completely on the ability of the implanted cells to differentiate toward mature cardiomyocytes. These cells must be able to reinforce the pumping activity of the injured heart in the absence of life-threatening arrhythmias due to electrophysiological incompatibility.

These conditions can only be met if the newly formed cardiomyocytes can integrate electromechanically with the host tissue while getting appropriate vascularization from host- or donor-derived vessels.

Together with the regeneration of the contractile component of the heart, attention must be paid to the search for novel methods to deliver appropriate vascularization to the ischemic areas and to the preservation of structures controlling the efficiency of blood pumping to the organism: the heart valves. Although fetal, neonatal, and adult cardiomyocytes have been tested for their actual ability to engraft diseased heart and improve its function (Dowell et al., 2003), most of the hopes in cardiac tissue regeneration relies on the possibility to use pluripotent and adult stem cells in targeted tissue engineering applications.

In this respect, the set-up of protocols to obtain reprogrammed pluripotent stem cells (Takahashi and Yamanaka, 2006), a discovery awarded with the Nobel Prize in Physiology or Medicine in 2012, represents the demonstration that such advances in knowledge and technology will eventually lead to novel approaches to the treatment of incurable diseases.

Unfortunately, notwithstanding a decade of intense investments, the results in terms of knowledge transferred to the clinical practice are very limited.

The present issue addresses the topic of cardiac tissue regeneration from the viewpoint of stem cell biologists and tissue engineers, seeking for the most appropriate source of cells to replace dead myocardium, improve the organ structure and function, as well as exploring the most suitable delivery system for such cells.

In the last couple of years a number of papers have been published on the ability of biomaterials and bio-constructs to drive the differentiation of stem cells for cardiac tissue engineering applications. Most of these studies used mesenchymal stem cells (MSCs) from bone marrow, adipose tissue or induced pluripotent stem cells (iPSCs). On the other hand, the contribution of cells derived from the heart tissues is here considered of outmost importance since these cells are believed to retain the potential to become highly differentiated cardiomyocytes.

## Looking for sources of contractile cells

Studies on the differentiation of embryonic cells into cardiomyocytes started in 1989 (Rudnicki et al., 1989). From that time, many candidates have been taken into consideration to substitute dead contractile cells, as myoblasts, hematopoietic lineages, and cardiac side populations.

While skeletal myoblasts can generate contractile cells that cannot couple electromechanically with the host myocardium (Forte et al., 2013), the other two stem cell subsets, hematopoietic stem cells (HSCs) and MSCs, are now universally recognized to retain no capacity to acquire the contractile phenotype (Balsam et al., 2004; Murry et al., 2004; Gnecchi et al., 2005; Siegel et al., 2012).

In the case of HSCs, after contrasting papers were published by the group of Piero Anversa and Charles E. Murry, the compelling conclusion that this bone marrow subset is not suitable for contractile cell regeneration was agreed (Orlic et al., 2001; Balsam et al., 2004; Murry et al., 2004).

The resistance of the other bone marrow-derived stem cell subset (bone marrow MSCs) as candidate to produce new cardiomyocytes has been even longer: after a seminal paper published by the group of Keiichi Fukuda in 1999 (Makino et al., 1999) it took more than 10 years to clarify that bone marrow mesenchymal progenitors have no cardiogenic potential.

A convincing investigation by Siegel and collaborators finally clarified that human MSCs are indeed able to acquire specific cardiomyocyte markers *in vitro*, while failing to show contractile functional features. When implanted in an *in vivo* model of rodent myocardial infarction, MSCs do not completely differentiate into functional cardiomyocytes, proving that BM-derived MSCs may not be used in cardiac regeneration (Siegel et al., 2012).

Additionally, the work of the group of Massimiliano Gnecchi recently explained the positive outcome of many pre-clinical and clinical trials entailing MSC administration, by showing that the predominant effect of implanted MSCs in the heart consists in the secretion of anti-apoptotic signals for the resident cells (Gnecchi et al., 2005).

The same mechanism has been recalled to explain the modest but positive results obtained by vessel-associated mesangioblast implantation in infarcted heart (Galli et al., 2005) and may be responsible of the positive outcome of the implantation of epicardially delivered adipose tissue-derived stromal cell (ADSC) sheets in a mouse model of dilated cardiomyopathy (Hamdi et al., 2013).

Other sources of mesenchymal progenitors are still credited of cardiogenic potential: the process of adipocyte dedifferentiation to produce mesenchymal progenitors has been known for some years, while only recently evidence that dedifferentiated adipocytes and adipose-derived mesenchymal cells can spontaneously acquire the contractile phenotype *in vitro* and help tissue healing *in vivo* was given (Jumabay et al., 2009). Interestingly, the use of this cell source for cardiac regeneration therapy would pose no availability issue, nor any immunological concern.

The choice of the cell and the construct to implant is of outmost importance. With the usefulness of MSCs as an easy source of cells for autologous implantation being questioned, the search for the best candidate as contractile cell substitute is still open. The use of cardiac resident progenitor cells—firstly being identified by Quaini et al. (2002) based on c-Kit+ expression and already being tested in a clinical trial (Bolli et al., 2011)—appears to be a promising alternative for cardiac tissue engineering. Nonetheless, compelling evidence of their efficacy *in vivo* still needs to be provided by independent research groups.

No matter the route of delivery of cells to the cardiac tissue and the cell source, small but significant improvements in terms of cardiac function have been repeatedly reported mainly through a paracrine effect (Mirotsou et al., 2011), while the goal of regenerating massive portions of cardiac tissue still remains elusive.

## Cardiac proto-tissues: trying to reproduce the complexity of the myocardium

The complexity of the myocardium resides in the number of cell types represented in it and in its highly hierarchical organization.

The ideal cardiac-specific scaffold would comply with cardiac muscle architecture, be deformable enough to sustain cardiac contraction, while being colonized by host vasculature and resorbed/degraded in due time. The biomaterials and scaffolds so far proposed for cardiac tissue engineering can be grouped into three categories: (1) scaffolds mimicking extracellular matrix (ECM) structure and/or the elasto-mechanical properties of the myocardium, (2) *in vitro* engineered cardiac patches, (3) three-dimensional (3D) scaffolds reproducing myocardial structural properties.

Culturing cells in three dimensions or on substrates displaying the mechanical properties of the myocardium has been deemed sufficient to prompt cardiac commitment but not complete maturation *per se* (Engler et al., 2006; Di Felice et al., 2013).

The attempts at designing scaffolds mimicking ECM structure and replicating the *in vivo* structural and mechanical features of the ECM, in order to provide stem cells with meaningful cues and comply with the architecture of the host tissue are far from being successful.

In an attempt to reproduce cardiac ECM stiffness, polyurethane acrylate (PUA) mold was recently used to pattern polyurethane (PU) and poly(lactide-co-glycolide) (PLGA) hydrogels via UV-assisted or solvent-mediated capillary force lithography (CFL) (MacAdangdang et al., 2014).

Alternatively, the combination of cell electrospraying and electrospinning to generate a solution of hydrogel and polyurethane blend was also proposed (Xu et al., 2014).

A natural approach to the issue of reproducing the ECM mechanics and topography was proposed by adopting human pericardium membranes to create 3D microporous scaffolds (Rajabi-Zeleti et al., 2014), or by using decellularized human myocardium as a biomaterial for cardiac tissue regeneration and engineering (Oberwallner et al., 2014).

Finally, hybrid biomimetic membranes were developed for cardiac tissue regeneration by binding the epitope sequences of laminin and fibronectin to poly(glycerol sebacate) (PGS) films (Rai et al., 2013). The same polymer has been successfully manufactured to obtain scaffolds displaying the anisotropic elasticity typical of the myocardium and promoting neonatal cardiomyocyte alignment (Engelmayr et al., 2008).

An example of cardiac constructs displaying the elasto-mechanical properties of the myocardium is given by the incorporation of thiophene-conjugated carbon nanotubes (T-CNTs) into polycaprolactone (PCL) electrospun meshes (Wickham et al., 2014) and using the polymer 3-hydroxybutyrate and 3-hydroxyvalerate (PHBHV). The combination of PHBHV with gelatin resulted in a 3D scaffold resembling the physico-chemical properties, micro-topography, and mechanics of the myocardium (Cristallini et al., 2014).

Given the superior performance in terms of survival and function of 3D co-cultures including endothelial cells (or their progenitors) and embryonic fibroblasts in skeletal muscle engineering (Levenberg et al., 2005), few attempts have been taken to create complex, multicellular engineered cardiac tissue constructs (proto-tissues) *in vitro* to be applied on damaged cardiac tissues.

For example, in this issue of Frontiers in Physiology, human MSCs have been used to provide interconnected vessel-like structures onto gelatin scaffolds, where pre-committed human cardiomyocyte progenitor cells (CMPCs) were then seeded (Pagliari et al., 2014).

Alternatively, a heterogeneous neonatal cardiac cell preparation was seeded onto fibrin gels until endothelial cells grew and an adequate contractile activity was registered (Tao et al., 2014).

Scaffolds may represent a very useful device to deliver cardiac stem cells to the ischemic or damaged heart. Besides the dream of generating personalized proto-tissues with finely controlled size and shape to match the particular need of the patient, the formulations proposed are far from reproducing the complexity of the cardiac tissue.

The use of medical devices may help researchers to reduce the number of implanted cells and to drive their differentiation through the use of embedded molecules or growth factors. And while the physico-chemical features of the scaffold proved to be important in prompting stem cell alignment and commitment, the role of the immune response in cardiac tissue engineering deserves more attention.

Although promising in terms of technology advancement, the extent to which these studies address the hierarchical organization of the myocardium is still far from being relevant. Cardiac tissue engineering holds the promise to revolutionize the future of heart disease treatment and the use of scaffolds may provide valuable 3D cues for stem cells to grow, differentiate, and distribute into an organized tissue.

## Concluding remarks

The common limitation of the studies so far proposed in cardiac tissue engineering remains the lack of a credible signature of complete cell maturation toward the cardiomyocyte phenotype. Most of the investigations showcased the expression of proteins typical of the cardiac tissue, and a spatial distribution or alignment of the cells inside the scaffolds. Some research groups were able to show a striation typical of myofibrils (Engelmayr et al., 2008), others the massive expression of cardiac troponins, formation of intercalated disks, and functional gap junctions (Di Felice et al., 2013; Cristallini et al., 2014). Unfortunately, the extent of differentiation shown is very far from the actual maturation required to rebuild the cardiac tissue complexity. Reproducing such complexity would require the formation of blood vessels, the connection of nerve terminations, the participation of cells from the immune system, fibroblasts, enterochromaffin cells, cardiomyocytes of the conduction system.

While waiting for bio-instructive scaffolds displaying a modular distribution of differentiative cues, the possibility to combine different cell subsets with distinct potential with 3D scaffolds reproducing the hierarchical structural complexity of the native tissue represents a valuable approach to generate complex cardiac constructs to treat cardiopathic patients.

### Conflict of interest statement

The authors declare that the research was conducted in the absence of any commercial or financial relationships that could be construed as a potential conflict of interest.
